# Postural Effects on the Mental Rotation of Body-Related Pictures: An fMRI Study

**DOI:** 10.3389/fpsyg.2018.00720

**Published:** 2018-05-23

**Authors:** Fangbing Qu, Jianping Wang, Yuan Zhong, Haosheng Ye

**Affiliations:** ^1^College of Preschool Education, Capital Normal University, Beijing, China; ^2^Department of Psychology, Nanjing Normal University, Nanjing, China; ^3^Center for Mind and Brain Science, Guangzhou University, Guangzhou, China

**Keywords:** embodied cognition, fMRI, effector-specific, in-rotation effect, mental rotation

## Abstract

This study investigated the embodied effects involved in the mental rotation of pictures of body parts (hands and feet). Blood oxygen level-dependent (BOLD) signals were collected from 18 healthy volunteers who performed mental rotation tasks of rotated drawings of hands under different arm postures. Congruent drawings of hands (those congruent with left-hand posture) evoked stronger activation in the left supplementary motor area (SMA), left precentral gyrus, and left superior parietal lobule (SPL) than did incongruent drawings of hands. Congruent drawings of hands (those congruent with right-hand posture) evoked significant activation in the left inferior parietal lobule (IPL), right SMA, bilateral middle frontal gyrus (MFG), left inferior frontal gyrus (IFG), and bilateral superior frontal gyrus (SFG) compared to that evoked by the incongruent drawings of hands. Similar methodology was implemented with drawings of feet. However, no significant differences in brain activation were observed between congruent and incongruent drawings of feet. This finding suggests that body posture influences body part-related mental rotation in an effector-specific manner. A direct comparison between the medially and laterally rotated drawings revealed activation in the right IPL, left precentral gyrus, bilateral IFG, and bilateral SFG. These results suggest that biomechanical constraints affect the cognitive process of mental rotation.

## Introduction

Previous studies have suggested that mental simulations during spatial transformation tasks share common temporal and kinematic mechanisms with those involved in actual task performance (Shepard and Metzler, 1971; Parsons and Fox, 1998). The mental rotation task, a specific spatial transformation task, requires subjects to mentally rotate 2-D or 3-D objects to an upright position and then judge their laterality (Parsons, 1994). Previous behavior results have suggested a general pattern that participants’ response time in the laterality judgment task are proportional to the time taken by the participants to physically move their hands in the position of the hand-stimuli (Parsons and Fox, 1998). This type of task requires that participants engage in object-centered reference frames; subjects must first rotate the representation of the object to a new position and then make a right or left laterality judgment.

Another type of spatial transformation task involves subjects judging the laterality of self-related stimuli, such as hands, feet, legs, faces, upper or lower limbs, and full body images (Valentine and Bruce, 1988; Zacks et al., 1999; Nico et al., 2004; Ionta et al., 2007; Takeda et al., 2009;Hetu et al., 2013; Tomasino and Gremese, 2016; Berneiser et al., 2018). This task requires subjects to use a self-centered reference frame (Shepard and Metzler, 1971; Parsons, 1987; Yoshizaki et al., 2007). Numerous studies have demonstrated that both core and general motor areas are involved in this type of task, specifically, the supplementary motor area (SMA), precentral gyrus, inferior parietal lobule (IPL), superior parietal lobule (SPL), and premotor cortex (PMC) (Hanakawa et al., 2007; Hetu et al., 2013). In addition, these behavioral and imaging studies demonstrated that the process of mentally rotating body parts shares similar or even identical brain activity with that which occurs during the actual movement of that body part (de Lange et al., 2005).

Numerous researchers have argued for the embodied nature of mental rotation by showing that body status (posture and configuration) influences laterality judgments of visual stimuli related to body parts (de Lange et al., 2006; Ionta and Blanke, 2009; Lorey et al., 2009; Curtze et al., 2010). Moreover, different variables have been explored in relevant experiments, such as perspective, stimulus orientation, laterality, and imagery movement complexity. Congruency between imagined movement and actual body posture has also been shown to influence motor imagery tasks (Fourkas et al., 2006). However, very few studies have explored whether congruency effects exist in mental rotation tasks involving body parts.

Until now, few researchers have focused on whether or not the postural effect is effector specific. Ionta and Blanke (2009) conducted a behavioral study to investigate whether the postural effect is effector-specific. In the experiment, participants were asked to place their right hands on their right knee and their left hands behind the back, and in the other condition the hand positions were reversed. The results showed that the reaction times (RTs) of the laterality judgments of right/left hands increased when the participants kept their right/left hands behind their backs, which suggested that the hands-only stimuli alone were modulated by the posture of the participants’ hands, while no hand-postural effect was found when using foot stimuli. This effect can be interpreted and termed as the “postural effect” in mental rotation tasks. However, the neural mechanisms underlying this effect must still be explored. In another study, de Lange et al. (2006) asked participants to judge the laterality of rotated pictures of hands under three body postures by pressing a response box with their toes. The results showed that the RTs of the hand laterality judgments followed the “biomechanical constraints” of left- or right-hand movements; i.e., when participants put their left or right arms in a biomechanically easy orientation (to judge the orientation of clockwise-rotated left-hand pictures, while their left hands were flexed clockwise, for example), the identification of the left or right hand in the opposite, biomechanically complex orientation (rotated orientations that were harder to actually achieve, for example, clockwise rotation of one’s right hand) was more difficult, as measured by increased RTs. These findings suggested that the posture of the upper limbs influenced the judgment of the rotated pictures of hands. However, in the study, the participants were asked to respond with their toes, with the underlying assumption being that regions involved in controlling the execution of big toe movements are not involved in controlling the mental rotation of hand/foot actions. However, such assumptions require further investigation in order to eliminate the influence of response execution.

The effects of biomechanical constraints on mental rotation have also been investigated (de’Sperati and Stucchi, 1997; Petit et al., 2003; Petit and Harris, 2005). The results suggested that the mental rotation of anatomically possible (or familiar) orientations is much faster than that of impossible (or unfamiliar) orientations. To demonstrate this, researchers used a motor imagery strategy that was grounded in biomechanical constraints (Parsons, 1987, 1994; Parsons and Fox, 1998; Creem et al., 2001; Tomasino and Rumiati, 2004; de Lange et al., 2006). Tao et al. (2008) explored the effects of biomechanical constraints (in-rotation effect) by classifying the orientation of pictures of hands into medial (toward the body midline) or lateral rotations according to their relation to the body. The results showed that medially rotated pictures of hands were recognized more quickly than were laterally rotated pictures. However, the neural mechanism involved in the in-rotation effect is still unknown and requires further neuroimaging evidence.

The present study investigated the above issues by using different orientations of pictures of hands and feet, while varying each subject’s arm postures. First, we used functional magnetic resonance imaging (fMRI) to identify brain activation differences between posture-stimulus congruent and incongruent conditions. We predicted that congruent postures would facilitate the judgment of congruent stimuli, manifesting as increased brain activity. Second, the addition of feet stimuli served to reveal the neural mechanisms underlying mental rotation and to clarify whether the mental simulation process is effector-specific. Third, we introduced a control task in our study to eliminate the influence of response execution by requiring the subjects to judge the laterality of left- or right-pointing arrows rather than left or right body parts. Finally, the classification of the stimuli into medial and lateral conditions allowed us to identify the brain networks specifically involved in the in-rotation effect, i.e., the facilitation of RTs when judging medially orientated (toward the body midline) drawings of hands versus laterally orientated drawings regardless of left or right hand (Tao et al., 2008). We expected subjects to have faster RT and stronger brain activation when judging medially rotated drawings than when judging laterally rotated drawings.

## Materials and Methods

### Ethics Statement

The Medical Research Ethics Committee of Jinling Hospital and Clinical School of Medical College at Nanjing University approved this study. Study participants provided written informed consent prior to their participation.

### Participants

Eighteen healthy college students (9 males and 9 females; age 23–27 years; all right-handed), with normal or corrected-to-normal visual acuity, participated in this study. The data from two participants were excluded due to technical problems.

### Stimuli

**FIGURE 1:**
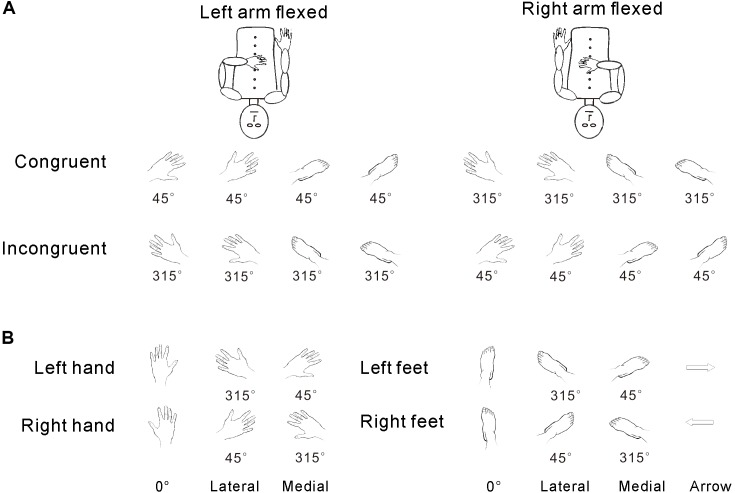
Congruency was defined in terms of the relative angle between the posture of the hand and the orientation of the stimuli (as described in the stimuli section and **A**). The medial/lateral distinction was defined relative to the physical angular position of the body (as described in the stimuli section and **B**), regardless of the relationship of orientation and hand posture.

The experimental stimuli included hand drawings of left/right hands or feet (**Figure 1**). Stimuli were presented in one of six clockwise (CW) orientations (45°, 90°, 135°, 225°, 270°, or 315°) (Tao et al., 2008). The upright orientation was defined as fingers/toes pointing upward (0°). First, to test postural effects, we divided the stimulus orientations into two groups based on the congruency between the stimulus and arm posture: (1) left arm flexed, A, congruent stimulus with 45°, 90°, and 135° orientations for drawings of the left hand/foot; B, incongruent stimulus with 225°, 270°, and 315° orientations for the drawings of the left hand/foot; and (2) right arm flexed, A, congruent stimulus with 225°, 270°, and 315° orientations for drawings of the right hand/foot; B, incongruent stimulus with 45°, 90°, and 135° orientations for drawings of the right hand/foot. The control stimuli were hand drawings of left or right-pointing arrows (see **Figure 1A**). Second, for the analysis of the effects of biomechanical constraint, the six orientations were separated into two groups: (1) medial (45°, 90°, and 135° orientations for the left hand/foot; 225°, 270°, and 315° orientations for the right hand/foot) and (2) lateral (225°, 270°, and 315° orientations for the left hand/foot; 45°, 90°, and 135° orientations for the right hand/foot) (see **Figure 1B**). All stimuli were projected onto a screen at the back of the MRI scanner and were viewed through a mirror in front of each participant’s face.

### Experimental Time Course and Procedures

Participants were asked to stay supine and motionless in the fMRI scanner and were unable to see their hands during testing. The task was to judge the laterality of the presented hand/feet drawings (left or right) by pressing the key of response-box that was firmly attached to their left or right toe. Participants were then instructed to mentally rotate their hands/feet in their self-perspective other than the object-based view point (third-person perspective) in test sessions.

**FIGURE 2:**
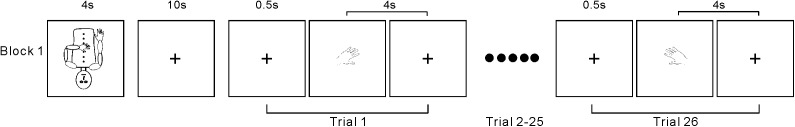
Time course of the first block. The other 19 blocks were the same as block 1.

The testing session consisted of 20 task blocks intermixed with 20 baseline periods. At the beginning of each block, a 4-s instruction picture was presented, requiring the participants to adopt one of the following two postures (**Figure 1A**): (1) left arm flexed toward the midline of the body and resting on the abdomen with the right hand resting naturally alongside the body or (2) right arm flexed and resting on the abdomen with the left hand resting naturally alongside the body. A marker was placed on the chest of each participant to designate the midline. The 4-s instruction period was followed by a 10-s baseline period, during which the participants fixated on a mark (+) on the screen. Next, participants completed a block of 26 trials, which included 24 body part trials (left/right hands/feet in 6 orientations) and 2 arrow trials (left- and right-pointing). Each trial started with a 500-ms fixation (+), followed by a hand drawing (**Figure 2**). Each participant made judgments by using his/her left or right big toe to press the left or right response key, respectively. The laterality and orientation of the drawings (hands × feet × arrow) were pseudorandomized. Based on previous research (Kosslyn et al., 2001), each picture was presented for 4 s and would disappear after this time period or after the participant responded. The picture was then replaced by a fixation (+) until the next picture appeared. In total, each participant performed 520 task trials [2 postures × 10 replications × (4 body parts × 6 orientations + 2 control stimuli arrows)] over a total of 43 min. After the experiment, the participants were asked about the use of ecocentric perspectives, rotation strategies, and their awareness of postural effects during their performance.

### Behavioral Analysis

Previous studies researching motor imagery effects on behavioral results (Cooper and Shepard, 1975) or physiological responses (de Lange et al., 2006) showed that stimulus view and in-rotation effects particularly influence RTs. Therefore, we investigated the influence of arm posture, stimulus congruency, and body part and stimulus orientation effects on RTs. RT was defined as the time between stimulus onset and the participant’s response (key press). Based on previous findings using hand stimuli ranges between 500 and 3500 ms (Kosslyn et al., 1998), responses faster than 500 ms or slower than 3500 ms were eliminated from the data analyses (total loss was 6% of the trials).

A 2 × 2 × 2 × 2 repeated-measures ANOVA was used to analyze the behavioral results, with stimulus congruency (congruent and incongruent), arm posture (left arm flexed and right arm flexed), stimulus orientation (medial and lateral), and body part (hands and feet) as independent factors and RT (ms) as the dependent variable. *Post hoc* simple *t*-tests were used to assess statistically significant interactions. An alpha level of *p* < 0.05 was used to determine statistical significance.

### fMRI Data Acquisition

All participants underwent functional scanning using a Siemens Trio 3T scanner at Jinling Hospital, Nanjing, China. Foam padding minimized the head motion of the participants. Functional images were acquired using a 90 single-shot, gradient-recalled echo-planar imaging sequence (repetition time = 2000 ms, echo time = 30 ms, and flip angle = 90°). Thirty transverse slices (field of view = 240 mm × 240 mm, in-plane matrix = 64 × 64, slice thickness = 4 mm, interslice gap = 0.4 mm, and voxel size = 3.75 mm × 3.75 mm × 4 mm) aligned along the anterior-posterior commissure line were also acquired.

### fMRI Data Analysis

The Statistical Parametric Mapping software (SPM8^1^) was used to conduct preprocessing of the functional images. The fMRI scans were initially corrected for temporal differences and head motion. No translation or rotational parameters in any given data set exceeded ±1 mm or ±1°. Functional images were warped to a standard stereotaxic space at a resolution of 3 mm × 3 mm × 3 mm using the standard Montreal Neurological Institute (MNI, Canada) echo-planar imaging template. Then, functional images were spatially smoothed with an 8 mm full width at half maximum Gaussian kernel.

For the first-level analysis, a general linear model (GLM) was computed for each session task and applied separate predictors for each participant. A boxcar function was convoluted using the hemodynamic response function. The boxcar function length covered the mental rotation interval of each trial section as well as the baseline period. Each condition included drawings of medial/lateral hands or feet and control stimuli (arrows). A random-effects two-sample *t*-test examined the significance of BOLD responses during the presentation of the experimental drawings (rotated drawings of hands) relative to that of the control stimuli (arrows) in order to distinguish the effects of the foot pressing.

The second-level analysis used *t*-tests to determine the influence of arm posture on mental rotation of the experimental stimuli. Four paired *t*-tests were conducted: (1) left arm was flexed, right arm was resting, congruent > incongruent stimuli (LH cong > incong); (2) right arm was flexed, left arm was resting, congruent > incongruent stimuli (RH cong > incong); (3) left arm was flexed, right arm was resting, congruent > incongruent feet stimuli (LH cong > incong); and (4) right arm was flexed, left arm was resting, congruent > incongruent feet stimuli (RH cong > incong). An additional paired *t*-test (medial > lateral) assessed the effects of biomechanical constraint on the mental rotation of body parts, irrespective of arm posture or stimulus type (hands of feet). Correction for multiple comparisons was performed using the AlphaSim program in the REST software (the parameters were as follows: individual voxel *P*-value = 0.001, 1000 simulations, FWHM = 4 mm, with mask), as determined by Monte Carlo simulations. Statistical maps of the two-sample *t*-tests were set at a combined threshold of *p* < 0.001 and a cluster size > 22 voxels, yielding a corrected threshold of *p* < 0.05.

## Results

### Behavioral Results

**FIGURE 3:**
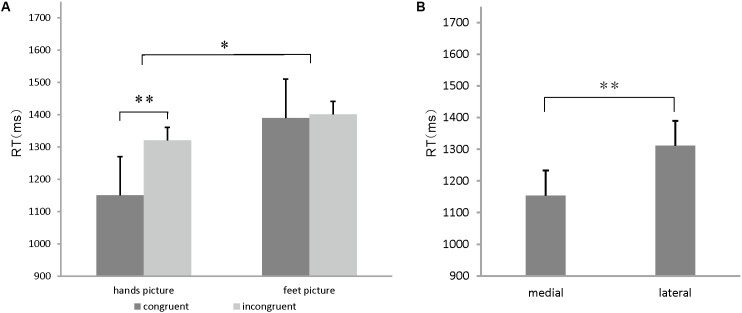
**(A)** Congruency effects on hands/feet stimuli. Mean reaction time (RT) for hands and feet stimuli for each congruent condition. Error bars represent the standard error of the mean. **(B)** In-rotation effect on hands/feet stimuli. Mean RT for stimulus judgment for each orientation condition. Error bars represent the standard error of the mean. ^∗^*p* < 0.05, ^∗∗^*p* < 0.01, ^∗∗∗^*p* < 0.001.

The ANOVA results showed significant main effects of the stimulus congruency [*F*(1,15) = 24.774, *p* = 0.000 < 0.05], stimulus orientation (medial/lateral) [*F*(1,15) = 9.47, *p* = 0.005 < 0.05], and body part [*F*(1,15) = 5.746, *p* = 0.03 < 0.05], a significant stimulus congruency by body part interaction [*F*(1, 15) = 13.24, *p* = 0.003 < 0.05] and a marginally significant stimulus orientation by body part interaction effect on RT [*F*(1,15) = 4.351, *p* = 0.054]. A slower performance in the incongruent conditions (1436 ms) than in the congruent conditions (1209 ms) accounted for the stimulus congruency effect (*p* = 0.000 < 0.05) (**Figure 3A**). Likewise, faster RTs to medial drawings of the hand/foot (1154 ms) than to lateral drawings of the hand/foot (1311 ms) contributed to the stimulus in-rotation effects (*p* = 0.005 < 0.05) (**Figure 3B**). A slower performance in response to the foot stimuli (1402 ms) than to the hand stimuli (1242 ms) accounted for the body part effect (*p* = 0.03 < 0.05). For the stimulus congruency by body part interaction, specifically, hand stimuli in the congruent condition were judged faster (1150 ms) than those in the incongruent condition (1320 ms). However, the RTs to foot stimuli did not differ between the congruent (1390 ms) and incongruent (1401 ms) conditions (**Figure 3A**). For the stimulus orientation by body part interaction, specifically, the RTs to hand stimuli in the medial orientation (1085 ms) were faster than the RTs to stimuli in the lateral orientation (1333 ms), whereas for the foot stimuli, no significant differences were found (1400 ms for medial orientation vs. 1471 ms for lateral orientation).

### Neuroimaging Results

#### Postural Effects

**FIGURE 4:**
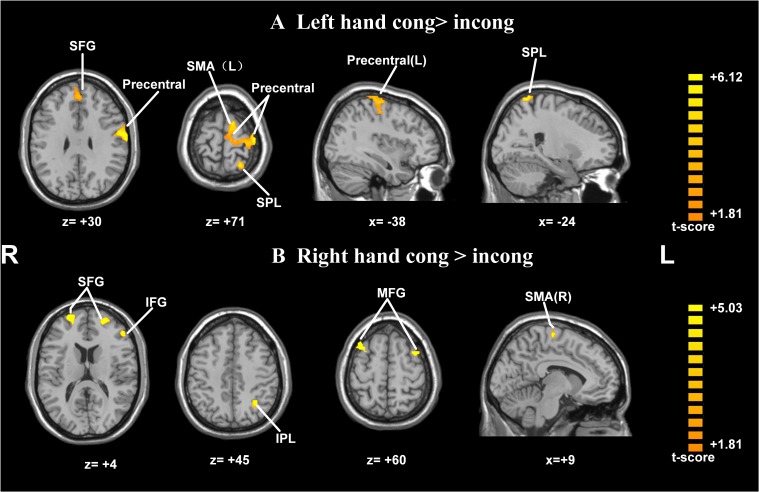
Statistically different *t*-maps between the congruent and incongruent drawings during left- or right-hand flexed conditions. (**A**, left forearm flexed: congruent > incongruent, left hand cong > incong; **B**, right forearm flexed: congruent > incongruent, right hand cong > incong) (paired *t*-test, *p* < 0.05, corrected). *t*-score bars are shown on the right. The numbers beneath the images refer to the MNI coordinates.

Postural effects were determined by four *t*-tests. The results showed that when the left forearm was flexed, the congruent drawings were associated with strong activation in the SMA, SPL, precentral gyrus, and superior frontal gyrus (SFG), all within the left hemisphere (*p* < 0.05, AlphaSim-corrected, *t* = 1.8125; **Figure 4A** and **Table 1**). When the right forearm was flexed, the congruent drawings of hands evoked strong activation in the IPL, SMA, SFG, inferior frontal gyrus (IFG), and middle frontal gyrus (MFG) (*p* < 0.05, AlphaSim-corrected, *t* = 1.8125; **Figure 4B** and **Table 2**). An additional analysis of the drawings of feet revealed no significant differences in terms of activation between the responses to congruent and incongruent drawings regardless of which arm was flexed.

**Table 1:** Significantly activated brain regions in congruent and incongruent drawings during left- or right-hand flexing conditions.

Contrast	Anatomical region	Hemisphere	Peak *T*-value	Stereotactic coordinates (MNI)			Voxels
LH cong > incong	Supplementary motor area	L	2.72	-9	-3	66	66
	Precentral gyrus	L	3.52	-57	-6	30	171
	Superior parietal lobe	L	3.31	-24	-60	72	38
	Inferior parietal lobule	L	2.23	-30	-54	45	40
RH cong > incong	Supplementary motor area	R	2.15	9	-21	66	39
	Middle frontal gyrus	L	2.57	-36	6	60	29
		R	2.84	33	9	60	119
	Inferior frontal gyrus	L	2.03	-51	36	12	87
	Superior frontal gyrus	L	3.16	-21	54	9	47
		R	4.09	27	57	9	50

Peak t-score and peak voxels are presented in MNI space. Cong, congruent condition; incong, incongruent condition; LH, left forearm flexed; RH, right forearm flexed.

**Table 2:** Significantly activated regions during the viewing of the hands and feet stimuli (medial × lateral, regardless of arm position).

Contrast	Anatomical region	Hemisphere	Peak *T*-value	Stereotactic coordinates (MNI)			Voxels
Medial > Lateral	Inferior parietal lobule	R	3	51	-51	54	139
	Precentral gyrus	L	4.37	-39	6	48	189
	Inferior frontal gyrus	L	3.29	-45	33	3	194
		R	3.26	57	21	6	191
	Superior frontal gyrus	L	1.97	-18	24	51	121
		R	2.86	30	27	51	266

*Peak t-scores and peak voxels are presented in the MNI space.*

#### In-rotation Effect

**FIGURE 5:**
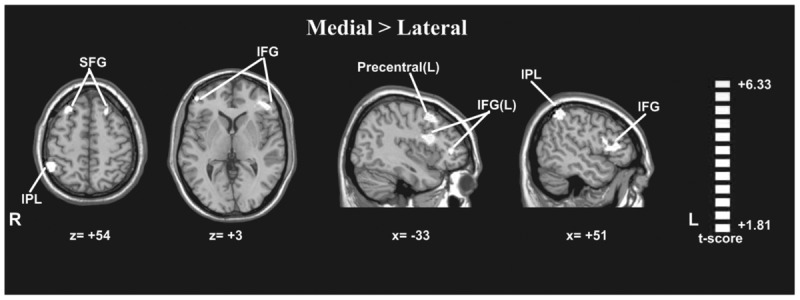
Statistically different *t*-maps between the medial and lateral drawings (paired *t*-test, *p* < 0.05, corrected). *t*-score bars are shown on the right. The numbers beneath the images refer to the MNI coordinates.

A paired *t*-test between the medial and lateral hand orientations (medial > lateral) showed increased BOLD signal (*p* < 0.05, AlphaSim-corrected, *t* = 1.8125) in the right IPL, bilateral SFG, bilateral IFG, and left precentral gyrus (**Figure 5**). These results suggested that orientation influences brain activity in areas related to mental imagery, such as the IPL, SFG, and IFG because the in-rotation trials require more spatial working memory retrieval and greater motor-related attention to daily actions than to stored actions relevant to the current presented drawings than do trials involving unfamiliar hand rotations (out-rotation).

## Discussion

The above results supported the idea of embodied cognition in several ways. First, our findings highlighted that a high-level cognitive process, such as mental rotation, was affected by body information, such as visual, proprioceptive, and somatosensory feedback. Sensory body effectors and previous body experiences influence the integration of current body status (Berlucchi and Aglioti, 1997). Second, the judgments of the drawings of feet remained unaffected by arm posture, suggesting that body sources influence cognitive processing according to an effector-specific rule. Third, biomechanical constraints influenced the integration of body information.

### Postural Effects

A large body of evidence supports the idea that the human brain contains specialized parietal-frontal circuits that are activated upon completion of a mental rotation task or when observing the rotational movement of others (de Lange et al., 2006; Lorey et al., 2009). Perspective and posture influence the mental rotation process, which includes the mental representation of the body and its context. In addition, the neural mechanisms underlying mental rotation are associated with the same brain activation responsible for an individual’s motor repertoire.

In our study, the congruency between arm posture and the imagined hand rotation led to faster RTs than incongruency did. The neuroimaging results showed that while the left hand was flexed in a clockwise orientation, the congruent drawings of hands evoked stronger activation in the left SMA, left precentral gyrus and left SPL than the incongruent drawings of hands did. When the right hand was flexed in a counterclockwise (CCW) orientation, the same comparison revealed significant activation in the left IPL, right SMA, bilateral MFG, left IFG, and bilateral SFG. This strong activation could be interpreted as facilitation resulting from the high amount of sensory input from the periphery (Shimura and Kasai, 2002). Previous studies using transcranial magnetic stimulation (TMS) in relation to motor imagery suggested that congruent postural signals induce greater excitability in the precentral gyrus than do incongruent postural signals (Vargas, 2004; Fourkas et al., 2006).

Our results also corroborated previous results, which found that the posture employed in mental rotation influences judgment (de Lange et al., 2006; Lorey et al., 2009; Wraga et al., 2009). Parsons and Fox’s (1998) PET study investigating mental rotation tasks of body part-related materials showed stronger activation in motor-related areas, including the pre-supplementary motor area (pre-SMA), inferior premotor cortex, superior frontal sulcus, and premotor cortex, than in other brain areas. These areas supposedly manipulate higher-order aspects of motor control, motor preparation and selection, action recognition and replication, spatial working memory, and guidance and execution. Furthermore, they also found that stronger activation was dominant in the left hemisphere, including in the SMA and IPL, two regions that are associated with planning for, guidance of, and attention to motor performance.

The parietal operculum, especially the IPL, is thought to function in higher-order somatosensory activity as well as in the integration of somatosensory information with other modalities (Caselli, 1993; Cipriani and Pandya, 1999; Servos et al., 2001). To complete the mental rotation task, participants need to process visual and somatosensory information from the primary somatosensory cortices and multisensory areas in the parietal lobe (Lorey et al., 2009). Other fMRI studies have also emphasized the role of the parietal lobe in action simulation and prediction, namely, the IPL, which is responsible for the prediction or expectation of a sensory stimulus (Carlsson et al., 2000). Therefore, our results were consistent with those of previous studies that suggested the involvement of the IPL in the embodied simulation process (Gallese, 2003, 2005; Keysers et al., 2004; Rizzolatti and Craighero, 2004).

The findings of the present study indicate that the drawings of feet were unaffected by upper limb posture and that congruent and incongruent drawings of feet elicited similar neural responses, which suggests that arm posture does not play a key role in mental rotation when the stimuli are not the same as the effector. The effect of arm posture modulation on neural activity during the mental rotation task was specific for upper limb body parts only and followed an effector-specific pattern.

### In-rotation Effect

One previous issue of debate in regard to mental rotation was what is actually simulated in the rotation process. Some researchers hold that mental rotation entails the simulation of a movement of one’s own body (Jeannerod, 1994), and this theory supports the notion of embodied cognition (Gallese, 2003), which insisted that body part-related mental rotation depends not only on the action requirements but also on the biomechanical constraints of one’s own body in space (de Lange et al., 2006). Other researchers suggest that the mental rotation process is an abstract implementation of general kinematic rules of biological motion and requires the knowledge of biomechanical constraints, not the actual biomechanical constraints (Pylyshyn, 2002).

The current study showed that medially rotated drawings of hands/feet led to faster RTs than did laterally rotated drawings, suggesting that the mental rotation of one’s body representation is faster under conditions of more familiar orientations (in-rotation effect) than under conditions of less familiar orientations. This result is similar to the findings of another study reported previously (Tao et al., 2008). The neuroimaging results revealed stronger activation in the right IPL, left precentral gyrus, bilateral IFG, and bilateral SFG in response to medially rotated drawings than to laterally rotated drawings. The IPL is thought to be involved in spatial working memory and attention to motor performance (Parsons and Fox, 1998). When presenting rotated drawings of body parts, which are physically possible and commonly encountered in daily life (in-rotation), spatial working memory and motor-related attention were engaged and matched with the current body position representation. Therefore, drawings in the in-rotation configuration evoked stronger activation in these motor-related areas than did drawings that were not in this configuration. This result suggested that biomechanical constraints affected the embodied cognitive process when participants imagined the rotation of the representation of their hands or feet.

## Conclusion

Our experiment showed that mentally rotating congruent drawings of hands leads to stronger activation of parietal and motor-related brain areas than incongruent drawings of hands. Our results supported the concept of the embodied nature of mental rotation of body parts by demonstrating that the inner presentation of one’s own body is actually simulated during the body part-related mental rotation. Moreover, biomechanical constraints influenced task performance in an effector-specific manner. Finally, increased parietal and prefrontal cortex activity associated with different arm postures revealed that proprioceptive factors play an important role in the cognitive process of mental rotation.

However, this study had some limitations that should be further explored. First, in this paper, we used only foot pressing as the response; hand pressing was not used. Although the confounding effect of foot pressing was distinguished by contrasting the hands or feet conditions with the control condition, it may be better to use a verbal response or a voice key. In addition, we mainly used the control task (arrows) to eliminate the influence of foot pressing on the neuroimaging results, and the behavioral data from the control task was not collected in the present study. Third, the classification of the medial and lateral orientations was based on their relation to the body midline; that is, a left hand flexed at 45°CW was classified as medially rotated, regardless of the hand posture (whether or not the left hand was flexed at 90°CW). This setting was not formerly used because no study has investigated the in-rotation effect and its relationship to body posture simultaneously. Therefore, this design was novel and needs further improvement. Previous studies also used human faces as stimuli for the mental rotation task; however, these studies did not consider the effect of emotion type (Valentine and Bruce, 1988; Civile et al., 2016), which would be an interesting subject in future studies on mental rotation.

## Author Contributions

FQ and HY completed the conception of the study. FQ and YZ collected fMRI data. FQ and YZ completed data analysis. FQ, JW, YZ, and HY drafted and complished the final manuscript.

## Conflict of Interest Statement

The authors declare that the research was conducted in the absence of any commercial or financial relationships that could be construed as a potential conflict of interest.

## Footnotes

1 http://www.fil.ion.ucl.ac.uk/spm

## References

[B1] Berlucchi G. Aglioti S. M. (1997). The body in the brain: neural bases of corporeal awareness. 20 560–564. 10.1016/S0166-2236(97)01136-3 9416668

[B2] Berneiser J. Jahn G. Grothe M. Lotze M. (2018). From visual to motor strategies: training in mental rotation of hands. 167 247–255. 10.1016/j.neuroimage.2016.06.014 27321046

[B3] Carlsson K. Petrovic P. Skare S. Petersson K. M. Ingvar M. (2000). Tickling expectations: neural processing in anticipation of a sensory stimulus. 12 691–703. 10.1162/089892900562318 10936920

[B4] Caselli R. J. (1993). Ventrolateral and dorsomedial somatosensory association cortex damage produces distinct somesthetic syndromes in humans. 43 762–771. 10.1212/WNL.43.4.762 8469337

[B5] Cipriani P. B. Pandya D. N. (1999). Cortical connections of the frontoparietal opercular areas in the rhesus monkey. 403 431–458. 10.1002/(SICI)1096-9861(19990125)403:4<431::AID-CNE2>3.0.CO;2-1 9888311

[B6] Civile C. McLaren R. McLaren I. P. (2016). The face inversion effect: roles of first and second-order configural information. 129 23–35. 10.5406/amerjpsyc.129.1.0023 27029104

[B7] Cooper L. A. Shepard R. N. (1975). Mental transformations in the identification of left and right hands. 104 48–56. 10.1037/0096-1523.1.1.48 1141835

[B8] Creem S. H. Wraga M. Proffitt D. R. (2001). Imagining physically impossible self-rotations: geometry is more important than gravity. 81 41–64. 10.1016/S0010-0277(01)00118-4 11525481

[B9] Curtze C. Otten B. Postema K. (2010). Effects of lower limb amputation on the mental rotation of feet. 201 527–534. 10.1007/s00221-009-2067-z 19902193 PMC2832871

[B10] de Lange F. P. Hagoort P. Toni I. (2005). Neural topography and content of movement representations. 17 97–112. 10.1162/0898929052880039 15701242

[B11] de Lange F. P. Helmich R. C. Toni I. (2006). Posture influences motor imagery: an fMRI study. 33 609–617. 10.1016/j.neuroimage.2006.07.017 16959501

[B12] de’Sperati C. Stucchi N. (1997). Recognizing the motion of a graspable object is guided by handedness. 8 2761–2765. 10.1097/00001756-199708180-00023 9295114

[B13] Fourkas A. D. Ionta S. Aglioti S. M. (2006). Influence of imagined posture and imagery modality on corticospinal excitability. 168 190–196. 10.1016/j.bbr.2005.10.015 16313979

[B14] Gallese V. (2003). A neuroscientific grasp of concepts: from control to representation. 358 1231–1240. 10.1098/rstb.2003.1315 12880530 PMC1693221

[B15] Gallese V. (2005). Embodied simulation: from neurons to phenomenal experience. 4 23–48. 10.1007/s11097-005-4737-z

[B16] Hanakawa T. Hosoda C. Shindo S. Honda M. (2007). Mental rotation of hands and feet involves somatotopically organized brain regions. 58:S60 10.1016/j.neures.2007.06.355

[B17] Hetu S. Gregoire M. Saimpont A. Coll M. P. Eugene F. Michon P. E. (2013). The neural network of motor imagery: an ALE meta-analysis. 37 930–949. 10.1016/j.neubiorev.2013.03.017 23583615

[B18] Ionta S. Blanke O. (2009). Differential influence of hands posture on mental rotation of hands and feet in left and right handers. 195 207–217. 10.1007/s00221-009-1770-0 19326106

[B19] Ionta S. Fourkas A. D. Fiorio M. Aglioti S. M. (2007). The influence of hands posture on mental rotation of hands and feet. 183 1–7. 10.1007/s00221-007-1020-2 17643238

[B20] Jeannerod M. (1994). The representing brain: neural correlates of motor intention and imagery. 17 187–202. 10.1017/S0140525X00034026

[B21] Keysers C. Wicker B. Gazzola V. Anton J. L. Fogassi L. Gallese V. (2004). A touching sight: SI I/PV activation during the observation and experience of touch. 42 335–346. 10.1016/S0896-6273(04)00156-4 15091347

[B22] Kosslyn S. M. Digirolamo G. J. Thompson W. L. Alpert N. M. (1998). Mental rotation of objects versus hands: neural mechanisms revealed by positron emission tomography. 35 151–161. 10.1111/1469-8986.3520151 9529941

[B23] Kosslyn S. M. Ganis G. Thompson W. L. (2001). Neural foundations of imagery. 2 635–642. 10.1038/35090055 11533731

[B24] Lorey B. Bischoff M. Pilgramm S. Stark R. Munzert J. Zentgraf K. (2009). The embodied nature of motor imagery: the influence of posture and perspective. 194 233–243. 10.1007/s00221-008-1693-1 19139856

[B25] Nico D. Daprati E. Rigal F. Parsons L. Sirigu A. (2004). Left and right hand recognition in upper limb amputees. 127 120–132. 10.1093/brain/awh006 14607796

[B26] Parsons L. (1994). Temporal and kinematic properties of motor behavior reflected in mentally simulated action. 20 709–730. 10.1037/0096-1523.20.4.709 8083630

[B27] Parsons L. Fox P. T. (1998). The neural basis of the implicit movements used in recognizing hand shape. 15 583–615.

[B28] Parsons L. M. (1987). Imagined spatial transformations of one’s hands and feet. 19 178–241. 10.1016/0010-0285(87)90011-9 3581757

[B29] Petit L. Harris I. (2005). Anatomical limitations in mental transformations of body parts. 12 737–758. 10.1080/13506280444000481

[B30] Petit L. S. Pegna A. J. Mayer E. Hauert C. A. (2003). Representation of anatomical constraints in motor imagery: mental rotation of a body segment. 51 95–101. 10.1016/s0278-2626(02)00526-2 12633591

[B31] Pylyshyn Z. W. (2002). Mental imagery: in search of a theory. 25 157–182; discussion 182–237. 10.1017/S0140525X02000043 12744144

[B32] Rizzolatti G. Craighero L. (2004). The mirror-neuron system. 27 169–192. 10.1146/annurev.neuro.27.070203.144230 15217330

[B33] Servos P. Lederman S. Wilson D. Gati J. S. (2001). fMRI-derived cortical maps for haptic shape, texture, and hardness. 12 307–313. 10.1016/S0926-6410(01)00041-6 11587899

[B34] Shepard R. N. Metzler J. (1971). Mental rotation of three-dimensional objects. 171 701–703. 10.1126/science.171.3972.701 5540314

[B35] Shimura K. Kasai T. (2002). Effects of proprioceptive neuromuscular facilitation on the initiation of voluntary movement and motor evoked potentials in upper limb muscles. 21 101–113. 10.1016/S0167-9457(01)00057-4 11983436

[B36] Takeda K. Shimoda N. Kato H. (2009). Motor imagery in mental rotation of hand pictures. 65:S169 10.1016/j.neures.2009.09.882 19484654

[B37] Tao W.-D. Huang X.-T. Zhang H. Feng S.-H. Liu Q. Tao X.-L. (2008). Observer’s hand orientation influences mental rotation of A hand stimulus. 41 10–25. 10.3724/SP.J.1041.2009.00010

[B38] Tomasino B. Gremese M. (2016). Effects of stimulus type and strategy on mental rotation network: an activation likelihood estimation meta-analysis. 9:693. 10.3389/fnhum.2015.00693 26779003 PMC4704562

[B39] Tomasino B. Rumiati R. I. (2004). Effects of strategies on mental rotation and hemispheric lateralization: neuropsychological evidence. 16 878–888. 10.1162/089892904970753 15200714

[B40] Valentine T. Bruce V. (1988). Mental rotation of faces. 16 556–566. 10.3758/BF03197057 3193887

[B41] Vargas C. D. (2004). The influence of hand posture on corticospinal excitability during motor imagery: a transcranial magnetic stimulation study. 14 1200–1206. 10.1093/cercor/bhh080 15142965

[B42] Wraga M. Flynn C. M. Boyle H. K. Evans G. C. (2009). Effects of a body-oriented response measure on the neural substrate of imagined perspective rotations. 22 1782–1793. 10.1162/jocn.2009.21319 19642881

[B43] Yoshizaki K. Weissman D. H. Banich M. T. (2007). A hemispheric division of labor aids mental rotation. 21 326–336. 10.1037/0894-4105.21.3.326 17484596

[B44] Zacks J. Rypma B. Gabrieli J. D. E. Tversky B. Glover G. H. (1999). Imagined transformations of bodies: an fMRI investigation. 37 1029–1040. 10.1016/S0028-3932(99)00012-3 10468366

